# Oxidative stress, DAMPs, and immune cells in acute pancreatitis: molecular mechanisms and therapeutic prospects

**DOI:** 10.3389/fimmu.2025.1608618

**Published:** 2025-08-20

**Authors:** Hanwen Chen, Yanhong Wang, Maddalena Zippi, Sirio Fiorino, Wandong Hong

**Affiliations:** ^1^ Department of Gastroenterology and Hepatology, the First Affiliated Hospital of Wenzhou Medical University, Wenzhou, Zhejiang, China; ^2^ School of the Second Clinical Medical Sciences, Wenzhou Medical University, Wenzhou, Zhejiang, China; ^3^ School of the First Clinical Medical Sciences, Wenzhou Medical University, Wenzhou, Zhejiang, China; ^4^ Unit of Gastroenterology and Digestive Endoscopy, Sandro Pertini Hospital, Rome, Italy; ^5^ Medicine Department, Internal Medicine Unit, Budrio Hospital Azienda USL, Budrio, Bologna, Italy

**Keywords:** acute pancreatitis, neutrophil, macrophage, oxidative stress, DAMPs

## Abstract

Acute pancreatitis (AP) is a gastrointestinal disease characterized by inflammation of the pancreas and is associated with high rates of morbidity and mortality. The pathogenesis of AP involves a complex interplay of cellular and molecular mechanisms, including oxidative stress, damage-associated molecular patterns (DAMPs), and the infiltration of various immune cells. This review aims to provide a comprehensive overview of the molecular mechanisms underlying AP, the role of different immune cells in its progression and potential therapeutic perspectives. Oxidative stress, characterized by an imbalance between reactive oxygen species (ROS) and the antioxidant defense system, plays a crucial role in AP. ROS not only contribute to cell necrosis and apoptosis, but also activate immune cells and perpetuate inflammation. DAMPs released from damaged cells activate the innate immune response by interacting with pattern recognition receptors (PRRs), leading to the recruitment of immune cells such as neutrophils, macrophages and dendritic cells. These immune cells further amplify the inflammatory response by releasing cytokines and chemokines. Neutrophils are among the first responders in AP, contributing to both tissue damage and repair, as well as the double-site sword effect of neutrophil extracellular traps (NETs). Other immune cells, including T cells, dendritic cells, mast cells and monocytes/macrophages, are involved in modulating the inflammatory response and tissue repair processes. The balance between pro- and anti-inflammatory immune responses is critical in determining the severity and outcome of AP. A table of targeted drugs or substances available in clinical trials is provided at the end of this paper, with the aim of providing available opportunities for clinical treatment. Nevertheless, precise targeted drugs are still urgently needed in clinical treatment, where more in-depth research is needed.

## 1 Introduction

Acute pancreatitis (AP), a pathological condition characterized by inflammation of the pancreas, is an increasingly important factor contributing to hospital admissions for gastrointestinal diseases (1). The significant morbidity and mortality rates associated with AP place a considerable burden on the healthcare system (2). Pancreatitis refers to the autodigestion of the pancreas in which premature activation of digestive enzymes plays an important role (3). The disease can be mild, affecting only the pancreas, or it can lead to systemic inflammatory response syndrome-associated extra-pancreatic organ failure and even death. There are many causes of the disease, including changes in alcohol and tobacco consumption patterns, obesity and diabetes (4). Severe acute pancreatitis (SAP), the most severe form of the disease, is associated with high morbidity and mortality (5). Currently, it is generally believed that AP initiates local inflammation and tissue damage, and then leads to systemic inflammatory response syndrome (SIRS) and even multiple organ failure (MOF) due to the inflammatory cascade (6). However, research on AP is far from complete and the specific mechanism of the disorder is still unknown.

Oxidative stress is one of the key metabolic changes in the immune response (7). In response to various stimuli or disease conditions, immune cells exhibit elevated levels of intracellular and extracellular Reactive Oxygen Species (ROS), which are associated with inflammatory molecular-cellular changes and effector responses. As signaling molecules, ROS are involved in the regulation of metabolic processes and inflammatory pathways. Dysregulation or overproduction of ROS may influence disease pathogenesis (8, 9). A range of cytokines are secreted by pancreatic acinar cells at the site of inflammation, leading to the subsequent infiltration of immune cells and either a pro- or anti-inflammatory response. Damage-associated molecular patterns (DAMPs) have a pivotal role in signal transduction and activation of the innate immune response. This occurs via their interaction with pattern recognition receptors (PRRs), which subsequently facilitate immune cell infiltration (10). Infiltrating immune cells include neutrophils, mast cells, dendritic cells, macrophages, natural killer cells (NK cells). They also include adaptive immune cells such as T lymphocytes and B lymphocytes. Immune cell infiltration is part of the inflammatory defense mechanism. Indeed, infiltration contributes to the recovery of AP. However, if the pathogen is not eliminated promptly, the resulting infiltration may have detrimental effects, such as exacerbating inflammation in immune cells through excessive autophagy and oxidative stress (11, 12). The potential negative effects may lead to the occurrence of distant organ damage and the development of multiple organ dysfunction syndrome (MODS) and SIRS. In the current review, we will discuss the effect of immune cells in the progression of AP.

Despite considerable advances in elucidating disease pathogenesis and identifying potentially effective therapeutic strategies by experimental means, there is still a lack of clinically validated medications.

## 2 Molecular mechanism

In this section, we will discuss two immune-related mechanisms governing AP progression from onset to recovery: oxidative stress and DAMPs. Oxidative stress (7) is associated with cell necrosis and apoptosis and triggers immune cells in the course of AP (9, 13). DAMPs are related to immune cell recruitment and tissue repair (14, 15).

### 2.1 Oxidative stress: ROS-driven AP injury

**Figure 1:**
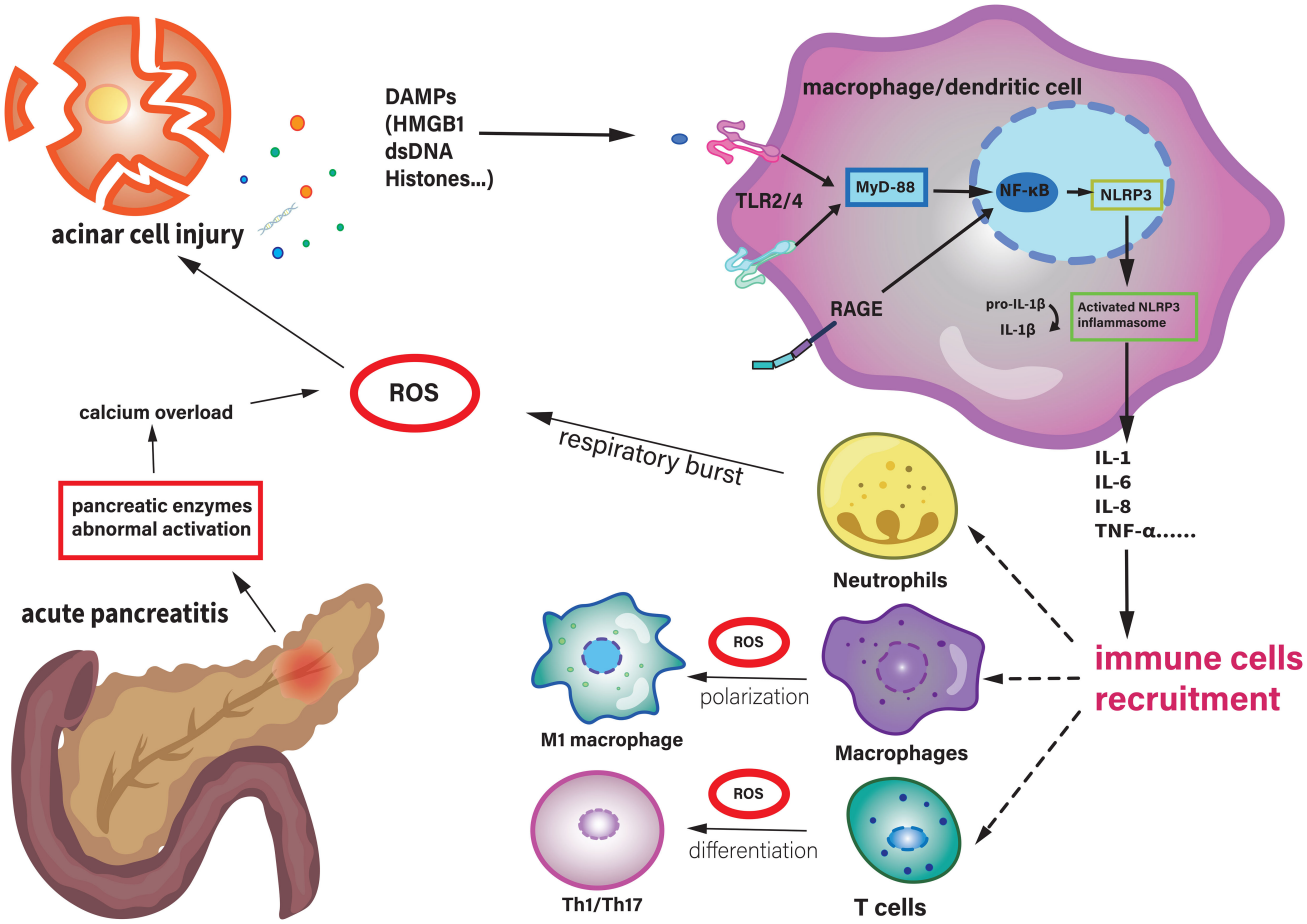
In acute pancreatitis, the abnormal activation of pancreatic enzymes leads to calcium overload within pancreatic cells, resulting in an increase in intracellular ROS. This also causes damage to acinar cells. Then, damage to pancreatic cells leads to the release of damage-associated molecular patterns (DAMPs, such as HMGB1, ATP, and mitochondrial DNA) into the extracellular space. These DAMPs are recognized by pattern recognition receptors (TLRs2/4) of macrophages and dendritic cells, activating NK-κB pathway and then NLRP3 inflammasome, to release IL-1β and other cytokines or chemokines. This further results in more immune cells recruiting to the site of injury, amplifying the inflammatory response and causing tissue damage. ROS can also be generated through the respiratory burst of neutrophils. ROS can promote the polarization of macrophages towards the M1 phenotype and the differentiation of T cells towards the Th1/Th17 direction. DAMPs, Damage-Associated Molecular Patterns; HMGB1, High Mobility Group Box 1; IL, Interleukin; MyD-88, Myeloid Differentiation Primary Response 88; NLRP3, NLR Family Pyrin Domain Containing 3; RAGE, Receptor for Advanced Glycation Endproducts; ROS, Reactive Oxygen Species; Th, T Helper Cell; TLR, Toll-Like Receptor.

Reactive oxygen species (ROS) are a key component of oxidative stress, and oxidative stress is a pathological condition caused by an imbalance between ROS and other oxidants and the antioxidant system in living organisms. ROS are an essential part of assessing whether the functions of a cell are running normally. In the development of AP, large numbers of immune cells are recruited to the organ. And the activation and proliferation of these types of immune cells are induced by ROS (16, 17). The relevant information is shown in **Figure 1**.

ROS are mainly generated at complexes I and III, due to electron leakage, which refers to electrons from electron transport chain (ETC) partially escaping, which results in the univalent reduction of molecular oxygen (O_2_) to superoxide anion (O_2_^-^·) and subsequent ROS (18). Cells possess a variety of antioxidant enzymes, such as superoxide dismutase, catalase, peroxidase, and glutathione systems, to regulate ROS levels, and overproduction of ROS exceeds the antioxidant capacity of cells, leading to oxidative stress. Oxidative stress can damage cell membranes, proteins and DNA, further exacerbating cellular damage and inflammatory responses (9, 19, 20). ROS can attack polyunsaturated fatty acids in cell membranes, leading to lipid peroxidation, which disrupts cell membrane integrity, resulting in cellular dysfunction and necrosis. As the stability of organellar membranes is compromised, for example, oxidized phospholipids (OxPL) are generated that can affect downstream biomacromolecules such as proteins, DNA, and lipids, which are integral parts of damage-associated molecular patterns (DAMPs), which we will discuss below (21).

Neutrophils release reactive oxygen species through respiratory burst, which could lead to necrosis (22). Apoptosis is genetically regulated, whereas necrosis is an uncontrolled mechanism of cell death (23). And it has been proven that the severity of AP is positively correlated with necrosis, and negatively correlated with apoptosis (24). The result of apoptosis is the clearance of cells from the body with minimal damage to the surrounding tissues, while necrosis leads to the spillage of cell contents into the surrounding tissues and their subsequent damage (25). At the same time, ROS can also promote the differentiation of T cells towards Th1 or Th17 (26).

Oxidative stress may also stimulate the activation of transcription factor and cause the excessive release of inflammatory mediators, including IL-1, IL-6, and TNF-α. Integrating our previous findings with established literature on p38MAPK signaling, we have identified that p38MAPK interacts with NADPH oxidase (NOX) to amplify reactive oxygen species generation, thereby establishing a self-perpetuating “ROS-p38MAPK” feedforward loop that exacerbates oxidative tissue damage. Based on this mechanistic insight, we hypothesize that targeted modulation of the p38MAPK pathway may attenuate inflammatory mediator expression and improve immune homeostasis, consequently mitigating oxidative stress and potentially ameliorating the clinical severity of acute pancreatitis (27–29). At different stages of AP, different concentrations of ROS produce different effects, in the acute phase, moderate amounts of ROS are beneficial for apoptosis, reduce necrosis and prevent severe pancreatic damage, but high concentrations of ROS cause pancreatic damage (30).

One experiment showed a significant increase in ROS levels in animal models of acute pancreatitis, which further promoted the activation and accumulation of inflammatory cells such as M1 macrophages and neutrophils, and also acted as a signaling molecule, leading to a significant increase in pro-inflammatory factors. The increase in ROS can disrupt the redox balance of the endoplasmic reticulum (ER), leading to ER stress and activation of the unfolded protein response (UPR), which in turn activates signaling pathways such as PKR-like ER kinase eukaryotic initiation factor 2 α (PERK-eIF2α) and activating transcription factor 6 (ATF6) and promotes the production of inflammatory factors (31).

Thus, ROS should not be considered simply as a toxic generation, but as a complex influencing factor. Certain substances that regulate ROS are listed in **Table 1**.

**Table 1:** Experimental therapeutic substance or drug for AP.

Invention type	Substance/drug	Model	Mechanism	Effect	References
regulate oxidative stress	Srxn1	mice	Inhibit activation of Cathepsin B and ROS induction	reduce ER stress and inhibit trypsin conversion	(31)
	Xanthohumol	mice	Decreased phosphorylated AKT(p-AKT) and phosphorylated mTOR(p-mTOR), inhibiting the activity of the AKT/mTOR pathway	restore autophagy function reduce inflammation and oxidative stress response	(167)
	Sitagliptin	C57BL/6 mice	activate the p62–Keap1–Nrf2 signaling pathway and promote the nuclear translocation of Nrf2	inhibit excessive autophagy and ROS production	(11)
	Epigallocatechin-3-g-allate	Balb/C mice	Significantly decrease the production of mitochondrial ROS	Inhibit NLRP3 inflammasome activation, reduce lung damage in AP	(168)
Inhibit NETosis	Irisin	C57BL/6J mice and their bone marrow neutrophils	directly combine with integrins αV/β5 and inhibit p38/MAPK pathway	reduce the formation of NETs	(159)
	PAD inhibitor Cl^‐^amidine	C57BL/6 mice	reduce the levels of histones H3 and H4, and the levels of DNA-histone complexes in plasma	reduce blood amylase levels and pancreatic edema, acinar cell necrosis, hemorrhage, and neutrophil infiltration	(169)
	GSDMD inhibitor disulfiram	platelet-specific Gsdmd knockout (KO) mice	directly binds to and inhibits GSDMD	reduce the formation of NET and inhibit pyroptosis	(170, 171)
	DNase-I	C57BL/6 mice	depolymerization of DNA in NET	clear NET and reduce neutrophil infiltration	(156)
Inhibit NF-κB pathway	Dexamethasone	Sprague-Dawley (SD) rats	Inhibit the expression of NF-κB, Bax and ICAM-1	Reduce the adhesion of leukocyte to endothelial cells	(172)
	Resveratrol	Balb/C mice	inhibit the inflammatory response mediated by IL-6-STAT3, NF-κB and PI3K pathway, inhibit TNF-α, IL-6	inhibit cell damage and necrosis, reduce the inflammatory cells infiltration to pancreas	(173, 174)
	Baicalein	C57BL/6 mice and RAW264.7 murine macrophages cells	Inhibit the degradation of IκBa and the phosphorylation of p65	Cut down the inflammatory cell infiltration and the production of pro-inflammatory factors	(175)
Regulate immune cells	Sivelestat	SD rats	Neutrophil elastase inhibitors, reduce lipase and amylase expression	reduce the inflammatory cell infiltration and histological damage	(176)
	Metformin	Zucker diabetic fatty (ZDF) rats,	Activate AMPK pathway, inhibit mTOR pathway, promote the transformation of macrophages into M2 type	Reduce the release of pro-inflammatory mediators, promote tissue repair	(177, 178)

### 2.2 DAMPs: from cellular release to systemic inflammation and repair in acute pancreatitis

Damage-associated molecular patterns (DAMPs) are endogenous danger molecules released by damaged or dying cells. DAMPs could activate the innate immune system by interacting with pattern recognition receptors (PRRs) (32, 33). They include ATP, the cytokine IL-1a, uric acid, the calcium-binding cytoplasmic proteins S100A8 and S100A9, and the DNA-binding nuclear protein high-mobility group box 1 (HMGB1) (34). DAMPs are recognized by PRRs, such as Toll-like receptors (TLRs) and cytoplasmic Nod-like receptors (NLRs), and also by non-PRRs, such as the receptor for advanced glycation end products (RAGE), CD44, integrins, and CD91 (15). As DAMPs are released locally and into circulation, they promote leucocytes infiltration and activation, which exacerbates pancreatic injury, systemic inflammation, and organ failure (35–37).

DAMPs normally reside inside the cell, but once the cell is damaged or dead, DAMPs were released into the extracellular space and then interact with PRRs. This leads to the recruitment of inflammatory cells and activation of adaptive immune responses. Excess DAMPs are also capable of activating signaling and sterile inflammation. DAMPs link local tissue damage and death to SIRS, leading to subsequent MOF and even death (14, 34). Interestingly, DAMPs are also involved in tissue repair (38). The detailed pathological roles of DAMPs in AP are summarized in **Figure 1**.

#### 2.2.1 HMGB1

HMGB1, one of the most prototypical DAMPs, is an abundant and highly conserved nuclear protein. By binding to and signaling through TLR4, HMGB1 could mediate the release of cytokines and tissue damage (39). It could also activate pancreatic macrophages to induce inflammatory genes including, TNF-α, IL-6, IL-1β, and MCP-1 (40). Downregulation of HMGB1 expression has been proven to inhibit Toll-like receptor 4 activation and enhanced protein kinase B (PKB) signaling, leading to subsequent inhibition of inflammation and apoptosis (41). Interestingly, the redox status of HMGB1 is involved in adjusting its activity. All-thiol HMGB1 can prompt autophagy by binding to RAGE, whereas disulfide-HMGB1 can exert pro-inflammatory effects by binding to TLR4. In the initial inflammatory phase, disulfide-HMGB1 activates immune cells and promotes the production of pro-inflammatory cytokines. During the late inflammatory response or tissue repair phase, reduced HMGB1 may promote cell migration and proliferation, contributing to the repair and regeneration of damaged tissues (42). Early blockade targeting HMGB1 could inhibit the release of HMGB1, which results in protection against injury in AP. On the contrary, intracellular HMGB1 helps to limit the release of nDNA (histones and DNA) and the subsequent recruitment and activation of inflammatory cells (43). Thus, HMGB1 appears to provide a wider therapeutic window in the treatment of AP.

#### 2.2.2 Histones

Histones are the basic proteins located mainly in the nucleus and could be divided into core histones including H2, H3, H4 and linker histones H1. Histones and DNA form a complex called a nucleosome. Under physiological conditions, the nucleosome is capable of regulating gene expression (44). For example, nucleosome can induce activation of cytosolic cyclic GMP–AMP synthase (cGAS), which results in synthesis of 2′3′ cyclic GMP–AMP (cGAMP). Then cGAMP binds to stimulator of interferon genes (STING). STING recruits TANK-binding kinase 1 (TBK1), promoting TBK1 autophosphorylation, and recruitment of interferon regulatory factor 3 (IRF3). In the end, the pathway could induce the release of IFN-β, which increases the function of macrophage and dendritic cells (45, 46).

Meanwhile, histones can also induce a hyperinflammatory response. When histone proteins are released into the extracellular environment, they activate the NF-κB pathway by recognizing TLR4, and the NF-κB pathway could induce the translation and transcription of NLRP3. Activation of NLRP3 started with oligomerization and recruitment of apoptosis-associated speck-like protein containing a CARD (ASC) and caspase-1, which led to the cleavage of pro-1β into activated cytokines (47–52). At the same time, the NF-κB pathway can also promote the production of a variety of pro-inflammatory cytokines and chemokines, and its overactivation can lead to amplification of inflammation and tissue damage (53).

### 2.3 TLR4/NF-κB: dual roles in AP inflammation and repair

TLR4 is localized on the plasma membrane and recognizes microbial components (54). Unlike other disease, the unique triggering factors of TLR4 are mostly endogenous, for example, histones as we have discussed above. In general, TLR4 and the subsequent pathways play a pro-inflammatory role in AP, however, there are also experiments showing that it also has a certain protective effect on AP. The research proved that the deletion of intestinal TRL4 could exacerbate AP by disrupting the intestinal flora and impairing the function of Paneth cell in mouse models, which leads to bacterial translocation (55). Therefore, although some experiments have shown that inhibiting TLR4 can improve AP (56, 57), we still need to view its effects dialectically.

Activation of TLR4 can induce the occurrence of autophagy, which is an intracellular degradation process in which damaged organelles, protein aggregates, and pathogens are enveloped by autophagosomes and subsequently degraded through fusion with lysosomes. TLR4 triggers autophagy via its downstream MyD88-dependent signaling pathway (58). In AP, the TLR4-mediated autophagy plays a dual role in exacerbation and resolution. On one hand, excessive autophagy may lead to cellular dysfunction, further exacerbating inflammation. On the other hand, moderate autophagy can clear damaged acinar cells, thereby alleviating inflammation. And one research also finds that autophagy and negatively regulate the excessive activation of TLR4 pathway in mouse models by degrading key molecules (such as MyD88 and TRAF6) within this pathway (59).

TLR4 is a pivotal receptor in the initiation and progression of AP. It primarily recognized DAMPs activating downstream inflammatory signaling pathway and promoting the release of pro-inflammatory cytokines, thereby exacerbating pancreatic injury and SIR (60). However, recent studies have revealed that in the later stages of AP, TLR4 may also contribute to inflammation resolution and tissue repair by activating cellular autophagy and suppressing excessive inflammation. This dynamic function suggests that TLR4 could serve as a potential therapeutic target of AP (58). Nevertheless, further investigation into its spatiotemporal regulatory mechanisms is required to balance pro- and anti-inflammatory effects, enabling more precise clinical intervention strategies.

There is also a close relationship between NF-κB and activation of trypsin. The production of pro-inflammatory cytokines that NF-κB pathway promote, induces dysfunction of lysosomes in pancreatic acinar cells, causing the leakage of lysosomal enzymes (such as cathepsin B), which leads to the activation of trypsinogen into trypsin (53, 60). The activation of trypsin leads to the necrosis of acinar cells, resulting in the release of pancreatic-specific DAMPs. This further amplifies inflammation through the TLR4/NF-κB pathway. These form a positive feedback loop, which is a specificity of AP, driving the progression of the disease (60). There is a study finds that in mouse models, the Bifidobacterium-derived metabolite, lactate, dampens macrophage-associated inflammatory responses both locally and systemically in AP, by suppressing NF-κB and NLRP3 inflammasome activation in a TLR4-MyD88- and NLRP3-Caspase1-dependent manner (61). However, the anti-inflammatory role of NF-κB is also being revealed. Studies have shown that the NF-κB activation protects acinar cells from inflammation-associated necroptosis, as well as NF-κB may reduce inflammation by limiting the processing and secretion of IL-1β (62), these two findings both indicate that it has a certain anti-inflammatory effect in the process of AP.

The combination of DAMPS and PRRS as an immune response to histiocyte injury such as acute pancreatitis can not only stimulate the accumulation of inflammatory cells and activate adaptive inflammatory responses, but also indirectly affect the severity of pancreatic inflammation and treatment methods through inflammatory cells.

## 3 Immune cells in AP

Immune cells are generated from multipotent hematopoietic stem cells (HSCs) residing in the bone marrow. And the differentiation of leukocytes is regulated by different sets of cytokines and cell-cell interactions. In the early stages of AP, the pancreas operates in a sterile environment, so pathogen-associated molecular patterns (PAMPs) are ineffective in recruiting or activating immune cells. However, the production of DAMPs by necrotized pancreatic acinar cells could trigger the activation of pattern recognition receptors (PRRs) on immune cells. This activation subsequently leads to the release of inflammatory mediators, facilitating the recruitment and infiltration of immune cells (63). Immune cell infiltration generally improves disease resolution as a defense mechanism. Platelets are also particularly involved in the process of the disorder. However, if there is an excessive accumulation of immune cells within a short period of time, or if they are not eliminated promptly, this can exacerbate pancreatic damage and contribute to systemic inflammation. This is due to the inflammatory response getting prolonged and intense (35).

### 3.1 Neutrophils

Neutrophils are polymorphonuclear white blood cells and one of the main responders to acute inflammation (64). As the first line of the innate immune response, neutrophils are well recognized as one of the key players during acute inflammation. Data from both experimental and clinical contexts indicate that the precise localization of neutrophils to the site of inflammation plays a pivotal role in the effective elimination of infection following infection. A prominent feature of AP is the infiltration of neutrophils into the pancreas (65). They are typically the first leukocytes recruited to the site of inflammation and acquire the ability to eliminate pathogens through a variety of mechanisms (66). Numerous studies have documented that neutrophil aggregation represents a critical element contributing to the development of AP. The process of neutrophil recruitment and activation involves a series of complex cascades. In inflammatory conditions, neutrophils have been recognized as harmful cells that inappropriately damage host cells. However, neutrophils are capable of recruiting to the sterile sites of inflammation, clearing dead tissue and cells and promoting tissue repair.

The inflammatory signals released by acinar cells mediate the recruitment and activation of circulating inflammatory cells, especially neutrophils. And excessive activation of these cells triggers intense local and systemic inflammatory responses (67). The secretion of platelet activating factor (PAF) induced neutrophils to release superoxide and cause degranulation. Toxicity was then diffused by the recruitment of neutrophils (68). During the development of AP, macrophage inflammatory protein-2 (MIP-2) was formed by macrophages and damaged acinar cells (69). MIP-2 regulates neutrophil chemotaxis and tissue neutrophils (70). The classical recruitment cascade of leukocytes has been extended to include capturing, rolling, adhesion, crawling, and transmigration (64). CXCL2 is the most effective stimulator for neutrophils to recruit and infiltrate (71). Neutrophils are free-flowing cells within the bloodstream. Capturing includes two closely related steps: primary capture, implying direct neutrophil/endothelial interaction, and secondary L–selectin–mediated capturing (72). Rolling relates to sialyl-Lewis^X^ and the neutrophil-expressed glycosylation-dependent receptor P-selectin glycoprotein ligand 1 (PSGL-1), which, following binding to E- or P-selectin (73). During the progress of rolling and deceleration, chemokine receptors on neutrophils, such as CXCR-2 and formyl peptide receptors, with their ligands present on the endothelium successfully interact, which triggers a series of signal responses finally leading to the conformational change of integrins and shear-resisting cellular adhesion (74). Integrins, such as those that interact with the endothelial cell ligand ICAM-1, cause neutrophils to stop rolling and remain on the endothelial cell surface (75). Adhesion is followed by crawling, which shows the movement of neutrophils along the endothelium. Mac-1 is the main regulator of intravascular crawling on ICAM-1 in the microvasculature (76). At the extravasation sites for neutrophils to transendothelial, happens by a transjunctional (paracellular) or a transcellular fashion. Inflammatory signals have been proved to enhance the way (77). Upon entering the interstitium, the neutrophil migrates in the so-called “amoeboid” form. Inflammatory chemokines or chemoattractants serve as the primary inducers of neutrophil polarity and deformational movement by activating intracellular signaling pathways through GPCRs (78). CXCL12 signaling has been shown to induce chromatin compaction by promoting H4K20 dimethylation, which is essential for neutrophil migration in challenging microenvironments (79). Once neutrophils are recruited to the site of infection, the pathogens are combated. In acute pancreatitis, the chemokine and cytokine cascades that accompany inflammation are functions of neutrophils that have been recognized for many decades (67). Direct evidence has shown that activation of trypsinogen to trypsin induces neutrophil infiltration at the sites through genetic knockout models. A curious discovery reveals that trypsinogen activation is thought to induce initial neutrophil infiltration into the pancreas, and subsequently the presence of neutrophils triggers further trypsinogen production (67). Similarly, MMP-9, found in neutrophils, has been suggested as a potential prognostic marker in pancreatitis and may be involved in neutrophil trypsin activation (80). Therefore, inhibiting the activation of trypsin, and thus the activation of neutrophils and damage to pancreatic tissue, could be a positive way to treat AP.

#### 3.1.1 NETs and AP

**Figure 2:**
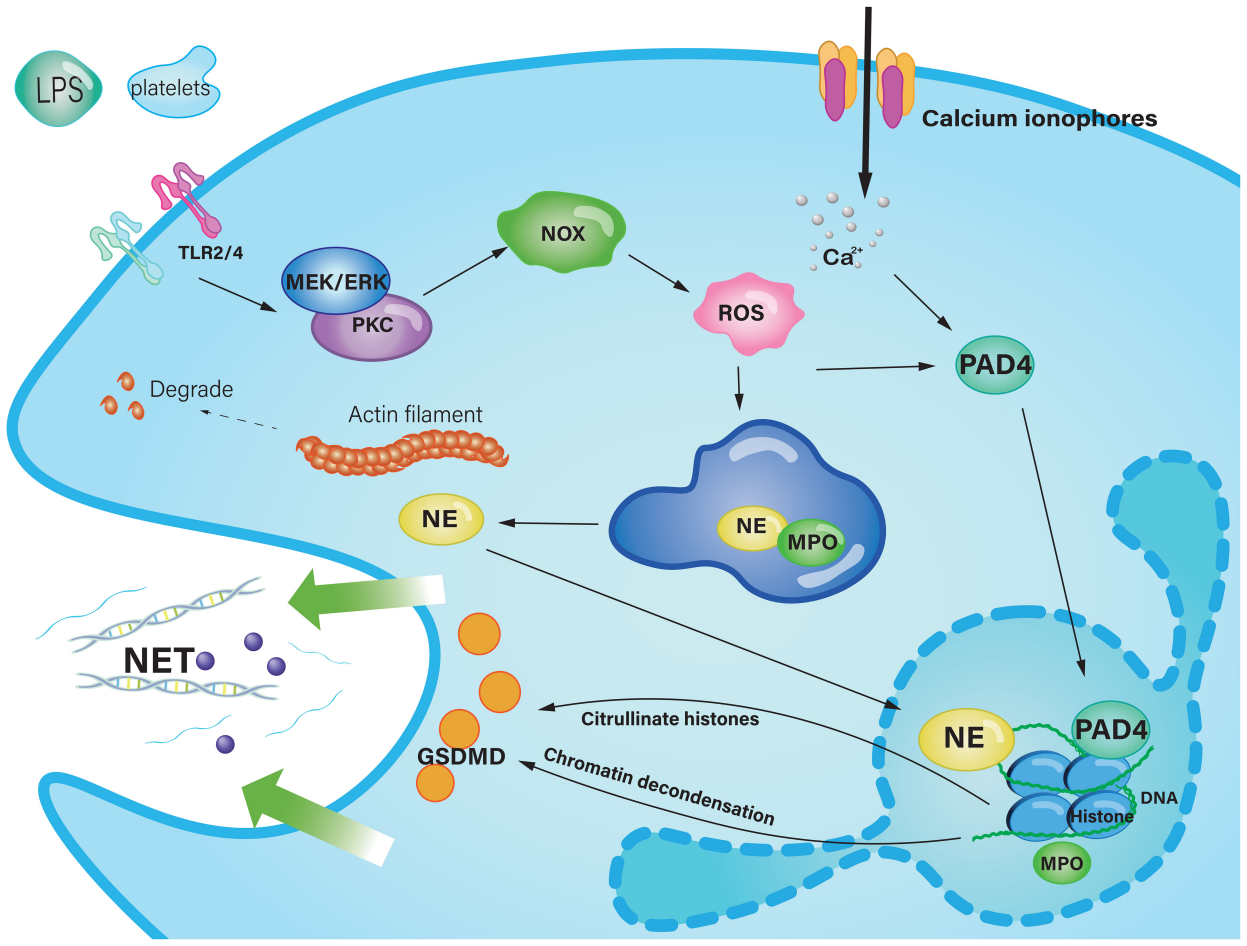
The classic pathway of NET formation in acute pancreatitis. Stimulus, such as LPS and platelets, trigger the MEK-pathway or PKC, resulting in the activation of NOX to provoke the production of ROS. ROS enter the azurophilic granule to liberate NE from the protein complex composed of MPO and NE. NE is transferred into the nucleus, subsequent to which core histones undergo proteolytic cleavage, culminating in chromatin depolymerization. Calcium ionophores cause a high concentration of Ca^2+^, which activate PAD4 to catalyze histone citrolination, destroy histone binding to DNA, and promote chromatin depolymerization. Decondensed chromatin DNA, histones, and cytosolic granzymes mix up, through the pore punched by GSDMD, they are effluxed to the extracellular space, and finally form NETs. Ca^2+^, Calcium ion; DNA, Deoxyribonucleic acid; ER, Endoplasmic reticulum; GSDMD, Gasdermin D; IL-8, interleukin 8;LPS, Lipopolysaccharide; MPO, Myeloperoxidase; NET, Neutrophil extracellular trap; NE, Neutrophil elastase; NOX, Nicotinamide adenine dinucleotide phosphate oxidase; PAD4, Protein arginine deiminase 4; PKC, Protein kinase C; ROS, Reactive oxygen species; TLR, Toll-like receptor.

Neutrophil extracellular traps are large web-like structures assembled on a scaffold of decondensed chromatin fragments as a skeleton and wrapped in histones, proteases, granules and cytoplasmic proteins (47). Furthermore, NETs induced the secretion of the proinflammatory chemokine IL-8 and the B-cell activating cytokine BAFF. Through pathways involving phosphorylation of Akt, ERK1/2 and p38, NETs induced neutrophil activation. NETs are a double-edged sword, as dysfunctional or excessive release can also lead to tissue damage (81, 82). To some degree, NETs-induced activation could exacerbate the inflammatory response, which could occlude the pancreatic duct and drive pancreatic inflammation (83). Currently, there are two ways of NET formation. The first is lytic NET formation, known as the cell death pathway and termed NETosis, which begins with nuclear delobulation and disassembly of the nuclear envelope and continues until loss of cell polarization, chromatin decondensation, and rupture of the plasma membrane. The second is a non-lytic form of NETosis that can occur independently of cell death and involves the secretion of nuclear chromatin accompanied by the release of granule proteins through degranulation (47). The formation of NETs via the NOX-dependent pathway begins with stimuli from pathogens or cytokines such as LPS (84). ROS are induced by MEK-extracellular signal-regulated kinase (ERK) signaling to trigger the myeloperoxidase (MPO) pathway or protein kinase C (PKC), simultaneously causing the assembly of the NADPH oxidase complex, leading to the generation of ROS (85). The latter enters azurophilic granules to dissociate the NE-MPO complex, NE then degrades the actin cytoskeleton in the cytoplasm to block phagocytosis (47). Also, the dissociated NE within the cytoplasm enters the nucleus to cleave the histone octamer, leading to chromatin densification. As a strong oxidant, MPO oxidizes tyrosine to tyrosyl radicals, which regulates enzyme activity in the cell signaling pathway (86, 87). Protein arginine deiminase 4 (PAD4), which synergistically catalyzes histone citrullination, impairs histone binding to DNA and promotes chromatin depolymerization, which is the basis for NET formation (81). Calcium ionophores directly activate PAD4 to induce NET release (86). In anticipation of NE and gasdermin D (GSDMD), the cell membrane breaks down and the depolymerized chromatin and cytoplasmic granzyme are effluxed into the extracellular space (88) (**Figure 2**).

With reference to AP, NETs regulate tissue damage and are involved in inflammatory damage, such as vascular pathological changes. NETs could promote vascular leakage and endothelial-to-mesenchymal transition through degradation of VE-cadherin and subsequent activation of β-catenin signaling (89). NETs contain an arsenal of cytotoxic proteases, including cathepsin G, proteinase 3, neutrophil serine protease 4, matrix metalloproteinase 9 (MMP9) and neutrophil elastase, which means that NETs could harm the endothelium while eliminating pathogens, for example, histones exert cytotoxic effects on acinar cells (90, 91). In addition, components of NETs could activate the intrinsic coagulation pathway to worsen the hypercoagulable state of blood (92, 93). Moreover, NETs in the pancreatic ducts could lead to catheter obstruction, which promotes the development of SAP (94). NETs directly regulate inflammatory cytokines or indirectly by modulating other immune cells (47). Inhibition of NET formation has been shown to attenuate tissue levels of CXCL2, which regulates neutrophil infiltration. NETs were also found to be a potent stimulator of Mac-1 expression and ROS formation in isolated neutrophils (91). NETs have also been found to activate other immune cells, such as B cells, antigen-presenting cells, and T cells (95). Thus, NETs may act as regulators of inflammation, which may be a promising target for future therapies (**Table 1**).

Genetic or pharmacological inhibition of neutrophil recruitment and infiltration could be a potential target therapy to alleviate AP. RING finger protein 128 (RNF128) inhibits neutrophil activation by binding to MPO and reducing its levels and activity (96). CXC chemokine inhibition with Evasin-3 reduces neutrophil-induced inflammation in the lung and pancreas, and also significantly reduces apoptosis in lung and pancreatic tissue (97). A Ras inhibitor (farnesyl thiosalicylicacid, FTS) significantly reduced MPO and serum amylase levels. Ras signaling also regulates neutrophil recruitment to the pancreas (98). Therefore, the mechanisms underlying neutrophil infiltration needed to be explored and served as intervention targets.

### 3.2 Dendritic cells

DCs are recognized as the most powerful antigen-presenting cells in the immune system (99). DCs include multiple subtypes, such as conventional DCs (cDCs), plasmacytoid DCs (pDCs) and monocyte-derived DCs (moDCs). cDCs originated from bone marrow pluripotent hematopoietic stem cells while moDCs are differentiated from monocytes at the site of inflammation (100). pDCs are mainly involved in anti-viral infections, and there are currently few studies on pDCs and acute pancreatitis, and it may be involved in the immune response in AP by producing IFN-I (101). In contrast, moDCs have poor migration capabilities but are capable of producing inflammatory cytokines and activating T cells to mediate inflammatory responses in inflamed tissues (100).

**Figure 3:**
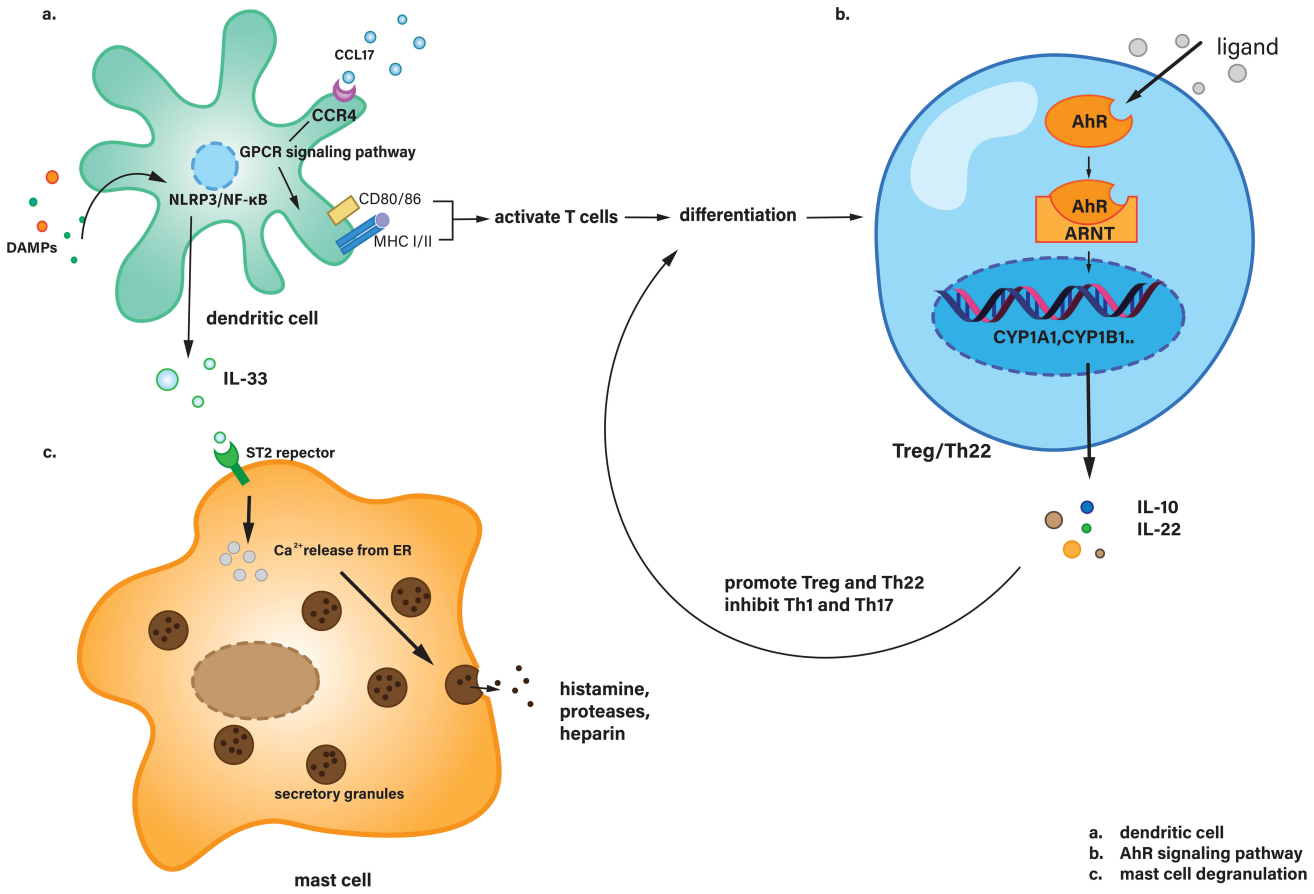
**(a)** Dendritic cells recognize CCL17 via CCR4 and activate T cells through downstream signaling pathways, while also secreting IL-33 in response to DAMPs stimulation. **(b)** In Treg or Th22 cells, the AhR signaling pathway is activated, leading to the secretion of IL-10 and IL-22, which regulate T cell differentiation. **(c)** Mast cells receive IL-33 signaling and undergo degranulation. AhR, Aryl hydrocarbon receptor; ARNT, Aryl hydrocarbon receptor nuclear translocator; CCL17, Chemokine (C-C motif) ligand 17; CCR4, C-C chemokine receptor type 4; CD80, Cluster of differentiation 80; CYP1A1, Cytochrome P450 family 1 subfamily A member 1; CYP1B1, Cytochrome P450 family 1 subfamily B member 1; ER, Endoplasmic reticulum; GPCR, G protein-coupled receptor; MHC, Major histocompatibility complex; ST2, Suppression of tumorigenicity 2 (IL-33 receptor).

Upon exposure to inflammatory stimuli, such as DAMPs, as discussed previously, immature DCs were activated and then triggered both adaptive and innate immune responses (102). Activated DCs promoted the recruitment of myeloid cells in the early AP (103). DCs were considered to promote the vitality of the pancreas during AP rather than damaging it. In type B coxsackievirus-induced acute pancreatitis, inflamed pancreatic acinar cells secreted CCL17, which was identified and combined with CCR4 on DCs. And the combination induced DCs to infiltrate the spot of inflammation. Then, it triggered further Th1 immune response (104), and protected the pancreas by reducing tissue injury. Another study showed that in the progression of AP in mouse models, acinar cells could express DC-SIGN, which could trigger the differentiation of naive CD4^+^ T cells into CD4^+^/IFN-γ^+^ Th1 and CD4^+^/IL-17A^+^ Th17 cells (the functions of Th17 and other T cells types will be discussed below) (105). Also, dendritic cells regulate the polarization direction of macrophages by secreting cytokines and chemokines. For example, IL-12 secreted by dendritic cells can promote macrophage polarization toward the M1 type and enhance their pro-inflammatory functions, whereas IL-10 secreted can induce macrophages to polarize to the M2 type and exert anti-inflammatory and tissue repair functions (106). This will be described in more detail in the following section regarding macrophage. During AP, DAMPs also activate DCs via PRRs, including TLR4 and the NLRP3 inflammasome. These damage signals trigger the NF-κB and MAPK pathways within DCs, promoting the transcription, synthesis and release of IL-33. IL-33 then binds to the ST2 receptor on mast cells, leading to their degranulation (107, 108). These will be explained in more detail in **Figure 3**.

### 3.3 T cells

Lymphocytes represent a specific type of white blood cell, produced by lymphoid organs with immune recognition functions. They are capable of participating in the body’s immune response. Lymphocytes can be subdivided into three main categories: natural killer (NK) cells, B lymphocytes (B cells), and T lymphocytes (T cells). This classification is based on the presence and characteristics of surface molecules, as well as the functions they perform. Humoral immunity and cellular immunity are mediated by B lymphocytes and T lymphocytes, respectively.

It has been demonstrated that, in addition to innate immune cells, T lymphocytes are present in the normal pancreas. During the course of acute pancreatitis, a continuous influx of T lymphocytes from the blood vessels results in a notable increase in the number of T lymphocytes within the pancreas (109).

#### 3.3.1 Activated regulatory T cells

It is well established that bacterial translocation and subsequent necrotic infection of the pancreas represent significant risk factors for severe disease and late death in AP (110).

Treg cells and Th17 cells are both lymphocytes. Treg cells are immune regulatory lymphocytes that secrete cytokines such as IL-10 and TGF-β, which compete with pro-inflammatory factors and inhibit the Th17 response. Th17 lymphocytes are T helper cells that secrete the IL-17 factor and typically elicit pro-inflammatory effects (111). An imbalance in the ratio of regulatory T (Treg) cells to Th17 cells has been implicated in the development of a number of immune-related diseases, with the potential to contribute to the onset of autoimmune disorders (112). The results of animal experiments indicate that regulatory T cells (Treg^-^) can be activated to inhibit the Th17 response, resulting in immunosuppression and disturbance of the duodenal barrier function. This, in turn, leads to an imbalance in the Treg/Th17 ratio in the duodenal mucosa, which affects intestinal mucosal leakage. Furthermore, the activation of Tregs can also inhibit the propria CD4^+^ T cells and duodenal CD8α^+^/γδTCR^+^ IELs, weaken the intestinal barrier function, and ultimately lead to the excessive growth of facultative pathogens, promoting duodenal bacterial transposition and pancreatic necrosis infection in severe pancreatitis (113).

Thus, in the context of immunotherapy, the role of Tregs in combating systemic inflammatory response syndrome may be considered a potential therapeutic target for preventing infection necrosis in acute pancreatitis.

#### 3.3.2 Activation of aryl hydrocarbon receptor

AhR is expressed in various T cell subsets, but the expression level varies between subsets. For example, expression was higher in Tregs, Th17 cells, and Th22 cells in mice, whereas expression was lower in Th1, Th2 cells, and initial T cells (114). As a transcription factor that regulates IL-22 expression, the activation of AhR has been demonstrated to protect against AP. Activation of AhR can induce the expression of IL-22, enhance IL-22 mediation, facilitate crosstalk between immune cells and pancreatic acinar cells, and reduce the number of IL-22^+^CD4^+^ T cells while increasing the number of IL-22RA1 during AP.

The classical AhR signaling pathway involves several key steps. Upon binding to its ligand, AhR translocate from the cytoplasm into the nucleus. Inside the nucleus, AhR forms a heterodimer with ARNT (aryl hydrocarbon receptor nuclear translocator). This AhR-ARNT complex then binds to xenobiotic response elements (XREs) in the promoter regions of target genes, such as CYP1A1 and CYP1B1, regulating their expression. These genes are involved in the metabolism and detoxification of various xenobiotics and endogenous compounds, playing a crucial role in cellular responses to environmental stimuli (115). Overall, AhR modulates the production of cytokines such as IL-10, IL-17, and IL-22, thereby influencing the interactions between T cells and other immune cells. For instance, IL-10 can promote the differentiation of T cells into Tregs (116, 117). The typical AhR signaling pathway is shown in **Figure 3**.

As a result, novel therapeutic targets for the treatment of AP may be identified through the regulation of AhR signaling pathways and AhR-mediated interactions between pancreatic leukocytes and epithelial cells.

### 3.4 mast cells

Mast cells are multifunctional immune effector cells derived from the bone marrow whose survival is critically dependent on cytokine signaling. These cells are involved in a variety of physiological and pathological processes, including but not limited to allergic responses, inflammatory pathways, immune homeostasis and tissue regeneration (118). In pancreatic tissue, mast cells are abundant and predominantly localized around vascular structures, lymphatic vessels and nerve fiber terminals within the pancreatic stroma. In addition, mast cells have a heightened sensitivity to even subtle environmental perturbations (119). They contain a substantial reservoir of mediators, including cytokines, histamine, proteolytic enzymes and platelet-activating factor, which can be rapidly released upon cellular activation, leading to degranulation. Upon activation by triggers such as IL-33 secreted by dendritic cells, mast cells undergo calcium influx, leading to the fusion of cytoplasmic granules with the cell membrane. This results in the rapid release of pre-formed mediators like histamine, proteases, and heparin, which increase vascular permeability and promote inflammation. Simultaneously, mast cells synthesize and release cytokines (e.g., TNF-α, IL-6), chemokines, and lipid mediators (e.g., prostaglandins, leukotrienes), amplifying the inflammatory response and recruiting other immune cells to the site of injury (120). This process significantly influences lymphocyte adhesion and chemotaxis, thereby initiating a spectrum of biological responses, including vascular dynamics within the organism. The specific distribution of mast cells and their rapid response to stimuli indicate that mast cells can form the body’s first line of defense against danger. The degranulation process is shown in **Figure 3**.

Mast cell activation is an early event in the pathogenesis of acute pancreatitis (121). It is known that in the early stages of acute pancreatitis, in addition to acinar cell destruction, injury factors stimulate the activation of mast cells, which activate and release a series of pro-inflammatory cytokines. Cytokines set off a chain reaction through a complex cytokine network, exacerbating pancreatic damage and inflammatory transmission. IL-1 cytokine system is activated in the early stages of acute pancreatitis, promoting destruction of pancreatic tissue and exacerbating the condition. As a member of the IL-1 cytokine superfamily (122), IL-33 can not only induce inflammation by using cell death as an alarm in certain situations, but also inhibit inflammatory signaling by regulating nuclear gene transcription (123, 124).

In experimental studies of acute pancreatitis and endothelial barrier dysfunction, Marwan Dib et al. (125) found that mast cells are involved in exudative pancreatic vascular disease, which occurs with increased permeability of pancreatic tissue capillaries. Plasma and inflammatory cells infiltrate into the pancreatic interstitium, activating various biochemical pathways and causing pancreatic damage and dysfunction. This damaging disease is linked to the activation and release of inflammatory factors such as histamine and leukotrienes by mast cells.

### 3.5 monocytes and macrophages

Macrophages are a type of innate immune cell that reside in the abdominal cavity (in close proximity to the pancreas) and in the tissues surrounding the pancreas. They can co-mediate and amplify the inflammatory cascade reaction in the AP process with neutrophils, lymphocytes and other immune cells, thereby influencing the severity of AP (53, 126–128). Prior to the development of AP within the body, a multitude of pathological pathogenic factors (such as biliary diseases, alcohol consumption, smoking, hyperlipidemia, and genetics) first result in a sustained elevation of Ca^2+^ within the cytoplasm of pancreatic acinar cells, premature expression of trypsinogen, activation of the NF-κB inflammatory signaling pathway, and a reduction in Ca^2+^ levels (129, 130). During the early phase of AP, stimulated pancreatic acinar cells secrete monocyte chemoattractant protein-1 (MCP-1/CCL2), which mediates the recruitment and migration of CCR2-expressing inflammatory monocytes (131). The CCL2 chemotactic gradient within tissues directs the polarized migration of monocytes toward pancreatic lesions, where infiltrating monocytes differentiate into M1-polarized macrophages. Damaged pancreatic acinar cells, early-infiltrated neutrophils, and M1 macrophages collectively amplify the secretion of pro-inflammatory chemokines and cytokines, thereby initiating a feedforward inflammatory cascade that exacerbates acinar cell necrosis (132). This leads to extensive destruction of pancreatic tissue and, in some cases, spread of inflammation to specific distant organs, which can result in multi-organ failure or intractable systemic inflammation. It is therefore possible to discuss the chemokines and inflammatory mediators that play an important role in the inflammatory cascade of AP in order to identify specific targeted drugs for AP.

#### 3.5.1 polarization and M1/M2 phenotypic switching

**Figure 4:**
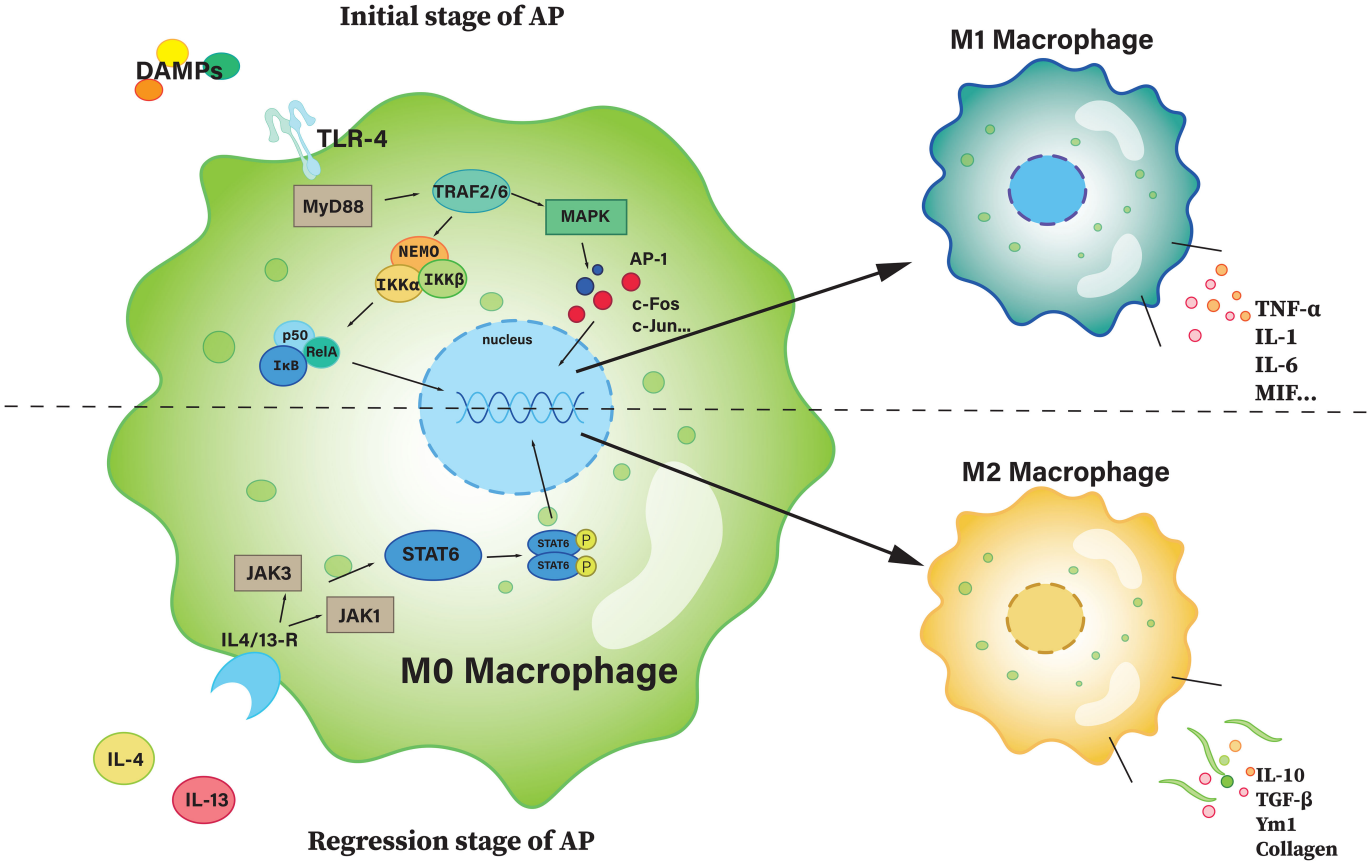
The polarization of M0 macrophage. The upper part of the figure is the path with a polarization of M1, and the bottom half is a path with a polarization of M2. AP-1, activator protein 1; c-Fos, cellular Fos; IKK, IκB kinase; JAK, janus kinase; MAPK, mitogen-activated protein kinase; MIF, macrophage migration inhibitory factor; MyD88, myeloid differentiation primary response 88; NEMO, NF-κB essential modulator; Rel A, v-rel avian reticuloendotheliosis viral oncogene homolog A (also known as p65); STAT6, signal transducer and activator of transcription 6; TRAF, TNF receptor-associated factor; Ym1, chitinase-like protein 3.

In the early phase of AP, necrotic pancreatic acinar cells become malfunctioning and release a significant number of cellular contents and debris, which induces the migration of inflammatory monocytes (CD11b+Ly6ChiCCR2+) from the bone marrow into the inflamed pancreas (127). TLR-4 is capable of recognizing extracellular information, stimulating and mediating intracellular signal transduction. During AP, once TLR-4 is activated, signaling is mainly through the NF-κB pathway and the MAPK-dependent pathway, and activation of these pathways promotes macrophage polarization towards a pro-inflammatory M1-like phenotype (7), while inhibition facilitating macrophage polarization to M2, the anti-inflammatory phenotype (126, 133). M0 can also recognize IL-4/13 and polarize towards M2 via the STAT6 pathway (134). The polarization pathways are described in more detail in **Figure 4**.

M1 promotes inflammation by secreting pro-inflammatory cytokines such as TNF-α, IL-1β and IL-6, as well as releasing inflammatory mediators that activate other immune cells, forming an inflammatory cascade to exert its pro-inflammatory effects (7, 134). On the contrary, M2 plays an anti-inflammatory role. Typically, during the remission phase of AP, with the suppression of pro-inflammatory pathways, macrophage polarization shifts toward M2. M2 secretes several anti-inflammatory cytokines, such as IL-10 and TGF-β, which can inhibit inflammatory responses and reduce tissue damage (135, 136). At the same time, M2 macrophages secrete extracellular matrix components (such as collagen and fibronectin) that promote tissue repair and regeneration. They also interact with M1 to inhibit its activation (134).

#### 3.5.2 Different chemokines and inflammatory mediators

During the initial phase of AP, the inflammatory response is characterized by robust monocyte infiltration into pancreatic parenchyma and peri-pancreatic tissues, followed by their differentiation into pro-inflammatory M1-polarized macrophages. The connected pattern recognition receptors on macrophages identify foreign pathogens and damaged cells and transmit information through kinase-dependent signaling pathways, thereby triggering the appropriate immune response and producing a substantial number of proinflammatory cytokines and inflammatory mediators. These include TNF-α, IL-6, IL-1β, PAF, etc., which play an important role in the progression of AP. Given the diversity of pro-inflammatory factors and the complexity of their mechanistic actions, we will focus our subsequent discussion on two critical mediators: TNF-α and MIF.

##### 3.5.2.1 Tumor necrosis factor-α

In the event of infection and subsequent inflammation of the pancreas, macrophages are able to respond rapidly to PAMPs and transcribe the TNF gene, which is already in a state of readiness, to rapidly produce TNF. As a result, TNF can be considered the first cytokine produced by macrophages in response to danger signals. The rapid response of TNF was associated with a relatively low proportion of histones with TNF promoters, with RNA polymerase II already located on the TNF gene. Furthermore, histones associated with TNF promoters contained the activation markers H3K4me and H3K9Ac, as well as signal-dependent transcription factor activation following receptor linking and localization and covalent modification of nucleosomes adjacent to TNF promoters (7, 137). This is consistent with other factors (138), as macrophages have been shown to have greater advantages in producing TNF than other cytokines. Upon activation, M1-polarized macrophages release TNF-α, which upregulates the expression of vascular cell adhesion molecules (VCAM-1/ICAM-1) on endothelial cells. This promotes leukocyte adhesion and endothelial activation, resulting in increased vascular permeability and subsequent pancreatic edema. Concurrently, TNF-α potentiates the secretion of IL-1 and IL-8, thereby enhancing leukocyte chemotaxis and directly amplifying pro-inflammatory responses. Collectively, these mechanisms exacerbate the early-phase inflammatory cascade in AP (139).

##### 3.5.2.2 Macrophage migration inhibitory factor

MIF is a crucial pro-inflammatory factor that can facilitate the local recruitment, proliferation and activation of macrophages in an inflammatory response, enhance macrophage adhesion and phagocytosis, stimulate the production of various pro-inflammatory cytokines and resist the immunosuppressive effect of glucocorticoids. In AP, the release of MIF from M1 macrophages is relatively significant. Under inflammatory stimulation, M1-polarized macrophages markedly upregulate the production and secretion of MIF. MIF binds to its cognate receptor CD74, augmenting MyD88-dependent signal transduction. This engagement activates the CD74-MyD88-IRAK4 axis, leading to NF-κB activation and subsequent transcriptional upregulation of pro-inflammatory cytokines, including TNF-α, IL-1β, and IL-6. Substantiating this mechanism, clinical observations demonstrate significantly elevated serum MIF levels in AP patients. Correspondingly, therapeutic administration of anti-MIF antibodies reduces mortality in experimental AP rat models (140). Therefore, it can be proposed that anti-MIF antibody is a potential starting point for the clinical use of drugs.

### 3.6 basophils and eosinophils

Basophils and eosinophils, two key granulocytes in the immune response, have been shown to play a role in various inflammatory responses that contribute to maintaining the body’s health (141). However, their involvement in the pathogenesis and progression of acute pancreatic inflammation has been less studied compared to other immune cells.

#### 3.6.1 basophils

Basophils mature in the bone marrow, circulate in the bloodstream and exhibit a range of biochemical and functional characteristics analogous to those of mast cells. They are recruited into tissues during inflammatory or immune responses in the body, and apoptosis occurs following their involvement in tissue reactions (142). Although studies on basophils in AP remain limited, existing evidence predominantly supports an IL-33-mediated recruitment pathway (143). The current understanding of the pathophysiology of basophils and type 1 autoimmune pancreatitis (AIP) has been discussed in the literature, but the mechanism of action in relation to AP remains unclear (144). It is worth noting that, in AP, basophils can release histamine through non-IgE-mediated pathways, such as IL-3-induced degranulation (145). The released histamine enhances vascular permeability in the pancreas, potentially contributing to pancreatic edema (146). At present, the role of basophils in AP remains unclear, and the interaction network between basophils and other immune cells still needs further exploration.

#### 3.6.2 eosinophils

IL-5 serves as a critical growth, differentiation and survival factor for eosinophils, while eotaxin-1 and eotaxin-2 function as eosinophil-specific chemokines that play pivotal roles in eosinophil recruitment and activation (147). In addition, in eosinophilic pancreatitis (EP), a rare type of pancreatitis, IL-5 is proven taking an important part in promoting the maturation, activation and inhibition of apoptosis of eosinophils (148). Vasoactive intestinal peptide (VIP) (149) and exotoxins (150) have been demonstrated to possess chemical properties that attract eosinophils, thereby facilitating their recruitment in the context of inflammatory conditions.

Eosinophils degranulation releases granule contents including major basic protein (MBP), which exhibit cytotoxic properties capable of directly damaging pancreatic tissue, leading to acinar atrophy and further infiltration of immune cells (147). In addition, eosinophils may further amplify the inflammatory response through interactions with other inflammatory cells, such as macrophages and T cells. Some clinical cases have demonstrated an association between eosinophils infiltration and pancreatic ductal inflammation or dysfunction. Mechanistic studies reveal IL-18 plays a pivotal role in eosinophil recruitment, it not only promotes eosinophil maturation and activation, but also directs their tissue-specific migration toward target organs including pancreatic ducts (151). Eosinophilic infiltration may also contribute to pancreatic vascular injury, manifesting as eosinophilic phlebitis and arteritis, which could potentially exacerbate pancreatic ischemia and inflammatory responses (151). Similarly, the relationship between eosinophils and the pathogenesis of AP is still not very clear at present, and further in-depth research is needed.

## 4 Treatment prospects for AP

Currently, most substances that regulate oxidative stress (OS) are primarily antioxidants, which exert protective effects by neutralizing excess free radicals. In various animal models of AP, these antioxidants have been demonstrated to mitigate OS-induced damage and promote tissue repair; for example, N-acetylcysteine (NAC) inhibits OS by scavenging excess ROS, enhancing glutathione levels and alleviating pancreatic tissue damage (152, 153). More substances/drugs are listed in **Table 1**.

Recent researches pointed out that therapeutic strategies targeting NETs focus on their lifecycle. The first is to inhibit NET occurrence by targeting key regulatory molecules to restrict NETs formation at the source (154, 155). The second is to accelerate NETs clearance by degrading the NET DNA backbone by DNase I or neutralizing their toxic components, to promote rapid removal of pathological NETs (156).

Immune system plays a crucial role in the course of AP. To date, therapeutic strategies targeting immune system regulation have become a hot topic. The main therapeutic approaches include inhibition of neutrophil activation and regulation of macrophage function (polarization). Also, inhibiting cytokines is a potential therapeutic strategy. A clinical trial shows that the administration of COX-2 inhibitors significantly reduced serum levels of IL-6 and TNF-α in patients with AP, accompanied by a marked decrease in the incidence of SAP (157). Another trial demonstrated that continuous renal replacement therapy (CRRT) effectively cleared immune biomarkers, including IL-6, IL-17, and HMGB1, from the serum of AP patients, yielding significant therapeutic benefits (158). Both clinical trials demonstrated the substantial therapeutic potential of immune therapy in AP.

Clinical translation remains a challenge, requiring precision in targeted therapies and long-term safety assessments. For example, Irisin has been proven to play a therapeutic effect in the AP model of mice (159). However, there are significant differences between human AP and mouse models: human AP is usually triggered by gallstones, alcohol or metabolic factors, presenting with complex systemic inflammatory responses and multi-organ failure, and with large individual variations; while mouse models are mostly induced chemically (such as caerulein) or through surgical methods (160), with more localized inflammatory responses and faster recovery, which means they are still lacking the disease background and immune characteristic with human. Future efforts should integrate multi-omics technologies and preclinical models to further optimize these intervention strategies.

In addition, research on gut microbiota and AP has developed rapidly in recent years. In the AP process, the intestinal microbiota destroys the intestinal barrier by activating NLRP3 inflammasomes, causing bacterial translocation and leading to infection and necrosis of pancreatic tissue, meanwhile, acute pancreatitis itself can also induce dysbiosis, forming a vicious cycle (161, 162). A clinical trial has also shown that by remodeling the structure of the gut microbiota, the intestinal barrier can be repaired and inflammation can be reduced (163).

With the development of new technologies, there are more ways to help us understand the pathogenesis of acute pancreatitis. For example, metabolic reprogramming in immune cells has shown that metabolic reediting plays a huge role in revealing the pathological mechanisms and treatment of pancreatic diseases such as pancreatic cancer (164, 165). Similarly, single-cell technology is also playing an important role, for example, one study using single-cell technology to track the chain of events from injury to death of pancreatic acinar cells (166). In conclusion, with the continuous emergence of new technologies, they are providing strong support for the development of more accurate diagnosis and treatment strategies.

## 5 Conclusion

In this review, we have discussed the interactions of oxidative stress, DAMPs and immune cells in AP. These interconnected pathways play pivotal roles in the pathogenesis and progression of AP, and offer promising therapeutic targets for intervention.

AP begins with the atypical activation of trypsin and subsequent pancreatic self-digestion, which can lead to a distressing progression of the disease. Despite numerous studies aimed at elucidating the precise pathophysiology of AP, the underlying mechanisms remain unknown. Targeted medicines have demonstrated efficacy in regulating the immune system. The overproduction of ROS during AP exacerbates pancreatic injury and systemic inflammation. Targeting oxidative stress through antioxidants or by enhancing endogenous antioxidant defenses holds significant potential to mitigate tissue damage. DAMPs, including HMGB1 and histone, amplify the inflammatory cascade via pattern recognition receptors. Therapeutic strategies aimed at neutralizing DAMPs or blocking the signaling pathways represent a novel approach to control inflammation. Immune cells, such as neutrophils, macrophages and T cells, play important roles in both inflammation and tissue repair. Suppressing excessive neutrophil activation, for example, could restore immune homeostasis and promote recovery.

In conclusion, we have provided a review concerning the role of oxidative stress, DAMPs, immune cells and their interaction in AP. We hope that this review will be useful and noteworthy for people working in these areas. A deeper understanding of the underlying molecular mechanisms is essential for the development of effective therapies. Further research should focus on translating these insights into clinical applications.

## Glossary

AhR: aryl hydrocarbon receptor
AIP: autoimmune pancreatitis
AP: acute pancreatitis
ASC: apoptosis-associated speck-like protein containing a CARD
ATF6: activating transcription factor 6
cGAMP: cyclic GMP–AMP
cGAS: cyclic GMP–AMP synthase
CRRT: continuous renal replacement therapy
DAMPs: damage-associated molecular patterns
EP: eosinophilic pancreatitis
ER: endoplasmic reticulum
ERK: signal-regulated kinase
ETC: electron transport chain
FTS: farnesyl thiosalicylicacid
GSDMD: gasdermin D
HMGB1: protein high-mobility group box 1
IRF3: interferon regulatory factor 3
MBP: major basic protein
MCP-1: monocyte chemoattractant protein-1
MIP-2: macrophage inflammatory protein-2;MMP-9, Matrix Metalloproteinase-9
MODS: multiple organ dysfunction syndrome
MOF: multiple organ failure
MPO: myeloperoxidase
NAC: N-acetylcysteine
nDNA: histones and DNA
NEMO: NF-κB essential modulator
NETs: neutrophil extracellular traps
NK cells: natural killer cells
NLRs: Nod-like receptors
OxPL: oxidized phospholipids
PAF: platelet activating factor
PAMPs: pathogen-associated molecular patterns
PERK-eIF2α: PKR-like ER kinase eukaryotic initiation factor 2 α
PKB: protein kinase B
PKC: protein kinase C
PRRs: pattern recognition receptors
PSGL-1: P-selectin glycoprotein ligand 1
RAGE: receptor for advanced glycation end products
RNF128: RING finger protein 128
ROS: reactive oxygen species
SAP: Severe acute pancreatitis
SIRS: systemic inflammatory response syndrome
STING: stimulator of interferon genes
TBK1: TANK-binding kinase 1
TLRs: Toll-like receptors
TNF-α: Tumor necrosis factor-α
Treg: regulatory T
UPR: unfolded protein response
VCAM-1: vascular cell adhesion molecule-1
VIP: Vasoactive intestinal peptide
XREs: xenobiotic response elements
